# Longevity

**Published:** 1896-04

**Authors:** F. H. Metcalf

**Affiliations:** Sacramento.


ARTICLE IV.

### LONGEVITY.

BY F. H. METCALF, D. D. S., SACRAMENTO.

Read before the California State Dental Association, July, 1895.

A man being asked in prayer meeting if he wanted to go to heaven, replied "No." "Do you want to go to hell?" asked the parson. "No." "Well, where do you want to go?" "Don't want to go anywhere; prefer to say right here in California." That man had a level head. It is safe to say that no person in possession of his faculties and in good bodily health wants to die. This is a beautiful world and we are put here to enjoy it in a legitimate way.

Two years ago I wrote a paper on "Ambidextrousness," believing it, among other things, conducive to longevity. Last year, on "Duty and Progress," finances and general care of the health. This paper is to be a continuation of the laws of diet, and shall refer more particularly to that much-abused organ, the stomach. Our moral and physical being depends so much upon it that it is deserving of our most careful consideration. An analysis of what goes into some stomachs in twenty-four hours would be startling, to say the least. If the stomach could talk, what a tale of woe it would impart. How often would it look up and say, "What's coming next?" Nothing is so conducive to long life, mental and bodily vigor as good digestion. The Bible says (Psalms, XC, 10th verse,) that the age of man is three score and ten years; still there are a few who reach it; though there are records of men vigorous at the age of 100. Again, in Ecclesiastes, it says "the grinders cease because they are few." But that was before the advent of dentistry and artificial teeth. Statistics show that the average length of life is on the increase. Is it not due in a measure to dentistry and the education of the masses regarding the laws of health? That few reach the allotted age is not due to heredity entirely, for with ordinary conditions of birth we have two grandfathers, four great-grandfathers, eight great-great-grandfathers, sixteen great-great-great-grandfathers; all responsible in a measure for our existence, and one could hardly throw a brick without its falling on the grave of an ancestor with a weak lung, stomach or kidney. Nature is bountiful, and has given most of us a good start, and, did we but take half care of ourselves, we should reach the age of 100 and still be in possession of much bodily and mental vigor. It is the pace that kills; not hard work. Gladstone and Bismarck furnish examples of long and active public life. I wish to give a few instances of exceptional longevity.

Henry Francisco, born in France, died in Whitehall, New York, in October, 1824, at the age of 135 years. John Gilley, born in Ireland in 2690, died at Augusta, Maine, in 1813, aged 123. Dr. Mussey, professor of anatomy at Dartmouth College, saw Gilley at the age of 118, and he was still active. He lived a bachelor until his 70th year, when he married, and became the father of eight children. Peter Zarten, near Temesvar, Hungary, died January 5, 1724, aged 185. Henry Jenkins of Yorkshire, England, lived to be 169 years of age. John Rovin and wife, of Temesvar, Hungary, died in 1741. He was 172 years at his death; his wife 164; their married life extended over a period of 147 years. J. Worchester, LL. D., gives a list of 98 persons in New Hampshire, all of whom were

100 or more years old. There were in 1858 in the United States 2,555 persons over 100 years old.

This proves that long life is possible and attainable. Rather an interesting experiment, to say the least. That longevity is more prevalent in New England than in other portions of the Union is not strange when we consider their mode of living, which is very plain. They are more methodical in their habits, and lead quiet, uneventful lives, they stick religiously to spring medicines, as the following anecdote shows:

"When snow and ice have gone," said the Sunday-school teacher, beaming on the boys, "and nature awakens from her long sleep, the tiny buds begin to appear, what do we have then? You may answer, Robert." "Sulphur and molasses" replied Robert earnestly. Whether there is any virtue in sulphur and molasses, aside from the druggist who deals in the mixture, I am not prepared to say.

People who live long, and retain their mental and bodily vigor, keep good hours and lead active lives. People rust out quicker than they wear out. Nutrition of the body can only come through activity of the body. Food, no matter how nutritious, is not easily drawn into the system, or assimilated, unless muscular activity is going on. Our lives, our dispositions and our mental capacity are regulated by what we eat, and there are few cases of ill-health that proper diet will not alleviate, but the trouble lies in the fact that it is cheap; the demand is for a cure that costs money. What a true saying: "Free prescriptions don't cure." How few people ever think or know of what elements the body is composed; and what food is required to maintain these according to climate, season, age, environment, etc.

The fourteen elements of the body are as follows: Oxygen, carbon, hydrogen, nitrogen, calcium, phosphorous, sulphur, sodium, chlorine and florine, iron, potassium, magnesium, silicum.

There are seventeen combinations of the fourteen elements of food: Water, gelatine, fat, phosphate of lime, albumen, carbonate of lime, fibran, floride of calcium, phosphate of soda, phosphate of potash, phosphate of magnesia, chloride of sodium, sulphate of soda, carbonate of soda, sulphate of potash, peroxide of iron, silica.

The processes of life in the body have the following divisions: First, the governing portion, the brain; second, the executive portion, the muscular system; third, the fuel, which, in a chemical sense, keeps up the supply of heat, which is the source of all activity and motion. Food must supply these three great divisions of the process of life in the proper proportion, or something will soon go wrong, though nature allows a wide margin.

Of the fourteen elements needed in the body, and which must be supplied in the food taken to satisfy the three great demands, vitality, strength and heat, I shall classify under the general terms, as follows (the words being used in their proper, and not chemical sense): The phosphates, in which phosphorus predominates, supply vitality or brain nerves and bone; nitrates, in which nitrogen predominates, supply the muscles with strength; carbonates, in which carbon predominates, supplying heat and making fat.

It is a lamentable fact that people in general know nothing of the nature of the food they eat, yet often wondering why they don't feel well. It is physically impossible to live for any length of time on food containing only a portion of the elements above mentioned, and keep well. "What we eat makes us what we are." It is our duty to learn in what proportion the elements of life are contained in the articles of daily food. This we should do for self-protection. As in most private houses, and particularly hotels, the *menu* does not vary with the seasons. Carbonates are as liable to predominate in hot weather as cold. The health of the guest is never taken into consideration. Wheat is one of the few cereals that contain the fourteen elements in nearly the right proportion. Bread is not the staff of life, unless made of wheat unbolted. In white flour we have little left but what is heat-producing; it impoverishes the blood, followed by fevers and headaches. How few mothers know to what extent the phosphates are demanded for growing children; and let me mention here that it is quite as important that children should have some food that requires an effort to masticate.

Most children's teeth suffer for want of exercise. Cracked wheat with good milk, lean meats not too tender; this, coupled with cleanliness, would do much to remedy existing evils. People living in San Francisco need more carbonaceous food than we do in Sacramento, where it is occasionally warm during the summer. Thousands of people ruin their stomachs from sheer ignorance of the laws of diet. "An ounce of prevention," etc. No profession offers better opportunities for spreading the truth than dentistry. Space will not admit my giving a complete list of foods, but I will mention a few that are particularly rich in the elements that produce heat, mental and physical activity. The best brain foods or phosphates are lean meats, fish, cheese, crabs, wheat, barley, oat meal, almond nuts, Southern corn, beans, peas, potatoes, figs and prunes. The best of carbonates, or heat-producers are fat meat, sugar, butter, rice, rye, chocolate, dates, buckwheat, Northern corn, white flour; excess in this branch is the cause of poor health, poor blood and bad skin. The best of the nitrogenous foods, or muscle-makers, are vermicelli, eggs, cheese, meats (particularly beef), Southern corn, salmon, beans and peas. Phosphate foods, for persons of strong mentality, and those who study much, cannot be too strongly urged. The best food is the cheapest.

Shakespeare says: "Dainty bits make rich the ribs, but bankrupt quite the wits." "Fat paunches make lean pates." How can a boy be expected to study who eats a breakfast in warm weather consisting, perhaps, of coffee, ham and eggs, white bread and buckwheat cakes. He complains of a tired feeling and is dosed for malaria, when all he needs is a change of diet and perhaps some wood to saw. Coffee and tea are responsible for many nervous disorders. Many persons injure their stomachs by taking their food in too concentrated a form; they need more food for waste, fruit and vegetable to distend the stomach, so that it may secrete the juices. Certain foods are receptive to certain parts of the body. The muscles of beef contain the same elements as human muscles, and are therefore adapted to nourish these. Meats of all kinds are more strengthening, taken from the part of the body having the most exercise. For instance, a rounk steak has superior nourishing qualities to a tenderloin. Crab and lobster contain a great quantity of brain and muscle food, but are so concentrated that they should not be eaten within several hours of retiring. People who believe in visions, dreams and other psychic phenomena, could account for it largely if they were reminded of what they ate before retiring. Swedenborg, the mystic, had his first vision after overloading his stomach. A piece of mince pie is harmless to look at, but is a terror taken before retiring. Crab salad, before retiring, will give most people a lively time in dreamland; but if one wants to enjoy Dante's Inferno take a Welsh rarebit at bedtime. Hearty food should never be taken at night, it causes restlessness and twitching of the muscles. Oysters are easily borne by the most delicate stomachs. They are composed of 12.6 nitrates, 2 of phosphates, balance waste. The abdominal portion is largely plain mud, and should not be eaten, as it is not pleasant to think of one's stomach being a receptacle for mud, and no doubt causes it to look like portions of the bay when the tide is out. To sum the matter up: on cold days eat heaters and muscle-makers; hot days, eat vitalizers and wastes, fruits; tired days, muscle-makers and phosphates; headache days, vitalizers; tea, coffee and condiments should be used sparingly. Think every day of what you eat; note how it agrees with you; study a table of foods and act accordingly; by practice one readily learns what will be conducive to health in his individual case. One need not deny himself anything wholesome, but study time, conditions and moderation.

Cheerfulness is producive of longevity, and should be cultivated. The seeds of cheerfulness are ambition, employment and an immediate purpose in life. Economy and thrift, simplicity in living and to be free from debt are wonderful aids to contentment, cheerfulness and long life. If a man earns $1 and spends 99 cents, result, happiness; if he spends $1.01 and earns 11, result, misery; difference between happiness and misery; 2 cents. Great riches, particularly if ill gotten, shorten life. An old man on his death-bed called his sons to his side and giving each a small bag of money, said: "Boys, the fortune that I leave you is small, but there is not a dirty shilling in the lot."

Oliver Wendell Holmes had about the right idea when he said. humorously:

I care not much for gold or land;
    Give me a mortgage here and there,
Some good bank stock, some note of hand,
    Or trifling railway share.
I only ask that fortune send
    A little more than I can spend.

Sadi said, speaking of riches, "Enough will carry you; more you must carry yourself."

We must have recreation also to reach the century mark. "All work and no play makes Jack a dull boy; and, if the work happens to be in a dental office, it might make him a weak man. Recreation gives moral and physical health, daring and endurance. The Duke of Wellington truly said: "The battle of Waterloo was won in the play-fields of Eton." We should drop our office cares, go to the country, fish, hunt and shoot, lose ourselves for the time being, inhale the wild flower air, which is invigorating to the mind as well as the body; inhale lots of air; practice abdominal breathing, whether walking, horseback riding or bicycling. One writer says: "The best thing for a man's inside is the outside of a horse;" and we may add, bicycle.

Presuming that from now on we lead such a life as shall bring us to a vigorous old age, what shall we see fifty years hence? What changes will have been wrought in persons, times and things? Let us hope that a common-school education, at least, will be compulsory with the masses. Let us hope that people will cease to speak to dentists of their trade, and want to know what D. D. S. means. We shall hope to see chairs of commonsense established in the schools and colleges of the land, filled by wise men who shall judge from contact and observation what calling or profession is best suited to young men entrusted to their care. Manual training we hope to see a prominent feature in the grammar and high school. The distinction between scholarship and usetulness will be better defined, and much that must be unlearned will give place to what may be applied. Money will not be considered the standard of excellence; children will not be pampered until their stomachs are ruined and their amusements exhausted, and an occupation purchased to keep them respectable. Enjoyments will be measured by capacity, body and mind will be one and inseparable, ingeniousness will be a social excellence.

We shall hope to see cooking and the proper selection of foods reduced to a science. All hotels should be compelled to employ, we will say, one professional educated cook, whose duty it shall be to see that no adulterated food goes into the kitchen, that foods are properly cooked and regulated according to season, climate, temperance, etc. The proper selection of food shall be taught in the school; education on that subject will be universal. Lectures will be given at short intervals on the care of the teeth and the general health in the different grades. Between the ages of ten and twenty the sexes will be separated in the schools, with teachers of their own sex. At the proper ages they will be instructed fully on the dangerous temptations and pitfalls incident to young manhood and womanhood. The cultivation of the graces will be a prominent feature. As a result we will see manly boys and womanly girls the rule.

Morals, it will be found, are largely emanations of the stomach, and men and women are good or bad as they are fed. Benefactions are generally traced to good dinners; crimes to bad ones. All classes will be gastronomically wise. People who live plain have fewer wants, therefore more leisure for social intercourse and cultivation of the mind. A limited amount of manual labor will be the rule for all, which shall increase our physical comfort and aid in our moral and intellectual development. Frankness in the office will be cultivated and practiced to the fullest extent. Dentists will never speak ill of one another, but all will strive for the greatest good to the greatest number.

That dress cuts some figure in the length of our lives I doubt not, but owing to the recent advent of the bloomer and the new woman, I have not the courage to prophecy or suggest reforms; but will venture the assertion that the longevity of women will not be diminished by the bloomer, and with her Trilby feet she will be enabled to wear her husband's fine boots with perfect ease; but let us offer a silent prayer that we men be still permitted to wear trousers.—*Pacific Stomatological Gazette.*

